# Uterine Prolapse in Pregnancy: Two Cases Report and Literature Review

**DOI:** 10.1155/2018/1805153

**Published:** 2018-10-22

**Authors:** Chunyan Zeng, Feng Yang, Chunhua Wu, Junlin Zhu, Xiaoming Guan, Juan Liu

**Affiliations:** ^1^Key Laboratory for Major Obstetric Diseases of Guangdong Province, Key Laboratory of Reproduction and Genetics of Guangdong Higher Education Institutes, Department of Obstetrics and Gynecology, The Third Affiliated Hospital of Guangzhou Medical University, No. 63 Duobao Road, Liwan District, Guangzhou, Guangdong 510150, China; ^2^Department of Ultrasound Medicine, Laboratory of Ultrasound Molecular Imaging, The Third Affiliated Hospital of Guangzhou Medical University, No. 63 Duobao Road, Liwan District, Guangzhou, Guangdong 510150, China; ^3^Division of Minimally Invasive Gynecologic Surgery, Department of Obstetrics and Gynecology, Baylor College of Medicine, 6651 Main Street, 10th Floor, Houston, Texas 77030, USA

## Abstract

Uterine prolapse complicating pregnancy is rare. Two cases are presented here: one patient had uterine prolapse at both her second and third pregnancy, and the other developed only once prolapse during pregnancy. This report will analyze etiology, clinical characteristics, complication, and treatment of uterine prolapse in pregnancy. Routine gynecologic examination should be carried out during pregnancy. If uterine prolapse occurred, conservative treatment could be used to prolong the gestational period as far as possible. Vaginal delivery is possible, but caesarean section seems a better alternative when prolapsed uterus cannot resolve during childbirth.

## 1. Introduction

Uterine prolapse is the descent of the uterus and cervix down the vaginal canal toward the introitus. Uterine prolapse during pregnancy is a rare event with incidence of one in 10000-15000 pregnancies, but this may be highly risky [1]. It can cause antepartum, intrapartum, and puerperal complication. Only a few cases of uterine prolapse during pregnancy have been reported and the efficiency of management varies from a conservative approach to laparoscopic treatment. We report two cases that simply benefit from a conservative management.

## 2. Presentation of Cases

### 2.1. Case 1

A 27-year-old Chinese woman, gravida 3, para 2, body mass index (BMI ) 17.20 kg/m^2^, visited our clinic with eight-week pregnancy in a prolapsed uterus on 4^th^ of September 2013. Pelvic examination revealed stage 3 pelvic organ prolapse (POP), with point C as the leading edge using the Pelvic Organ Prolapse Quantification (POPQ) examination (Aa+3, Ap+3, Ba+6, Bp+6, C+6, D+2, gh 4.5, pb 2, tvl 9 ). Her prolapsed uterus could be restored to pelvic cavity within bed rest. It was more serious while standing or walking. Hospitalization was recommended for this pregnant woman, but she refused and she waited at home for delivery.

Her previous pregnant record was as follows: a dead female baby was induced at the 30^th^ week of gestation during her first vaginal delivery in 2003, puerperium was uneventful, and two days after delivery, she was discharged in good health. She had her second vaginal delivery, after 38^+3^rd week of gestation and seven-hour labor in 2007; a 2800 g alive baby boy was delivered, with Apgar scores of 10/10. Pelvic examination revealed stage 3 POP using the POPQ examination (Aa+3, Ap+3, Ba+6, Bp+6, C+6, D+2, gh 4.5, pb 2, tvl 9) at the 36^+3^rd week of gestation in her second pregnancy. No special examination or treatment was executed before and after childbirth. However, the prolapsed vaginal mass was spontaneously restored after childbirth.

The woman presented to our hospital again with premature rupture of membrane (PROM) in labor at 39^+6^th week of gestation with an irrestorable uterine prolapse for 8 months on the 8^th^ of May 2014. Pelvic examination revealed stage 4 POP using the POPQ examination (Aa+3, Ap+3, Ba+9, Bp+9, C+9, D+5, gh 4.5, pb 2, tvl 9 ) and it revealed that prolapsed uterus was in size of 20×20 cm, pink, hyperaemic, and edematous but not ulcerated. The cervical canal did not subside, internal orifice of cervix did not open, amnionic vesicle has been broken, and regular contraction was seen. A series of transabdominal ultrasonographic examinations showed a normally developing fetus in the longitudinal position in the uterine cavity, isthmus uteri was 64 mm and it was partially extruded outside the vulva which was protruding from the perineum about 64×68 mm, and the boundary was still clear, and with cervical oedema. Emergency caesarean delivery was decided and an alive boy baby weighting 2480 g, with Apgar scores of 10/10, was delivered. We used Magnesium Sulfate Solution to nurse the prolapsed uterus. Three days postpartum, the prolapsed uterus was in size of 10×10 cm. On the seventh day postpartum, the prolapsed uterus was in size of 7×5 cm, and it was restored inside the pelvic cavity after manual reposition. Pelvic floor three-dimensional ultrasound indicated that residual urine was 40 ml, cervical length was 5.6 cm and internal orifice cervix was dilated, bladder neck displacement was 15 mm, posterior angle of bladder was 180 degree, and hiatus of levator antimuscle was 32 cm^2^. She was discharged on the eighth days postpartum. A telephone postpartum follow-up on the 14th day showed that there was no lump prolapse when the patient was standing or walking. But when the abdominal pressure increased, such as when squatting and defecating, prolapsed vaginal mass could be palpable, with size of 2 cm × 1 cm. 42 days after childbirth, she refused regular postpartum examinations for personal reasons.

### 2.2. Case 2

A 33-year-old Chinese woman, gravida 2, para 1, BMI 20.70 kg/m^2^, noticed a protrusion in size of 2 × 1 cm from her vagina at 13th week of gestation in 2015. Her first pregnancy resulted in one uncomplicated spontaneous vaginal delivery in 2009; the newly-born baby weighted 3000 g. There was neither history of pelvic trauma or prolapse, nor any stress incontinence during or after the first pregnancy.

**Figure 1:**
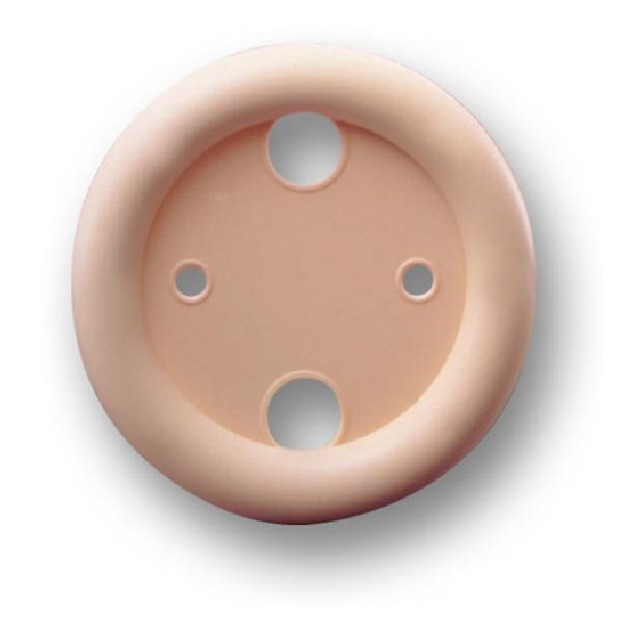
Ring pessary.

**Figure 2:**
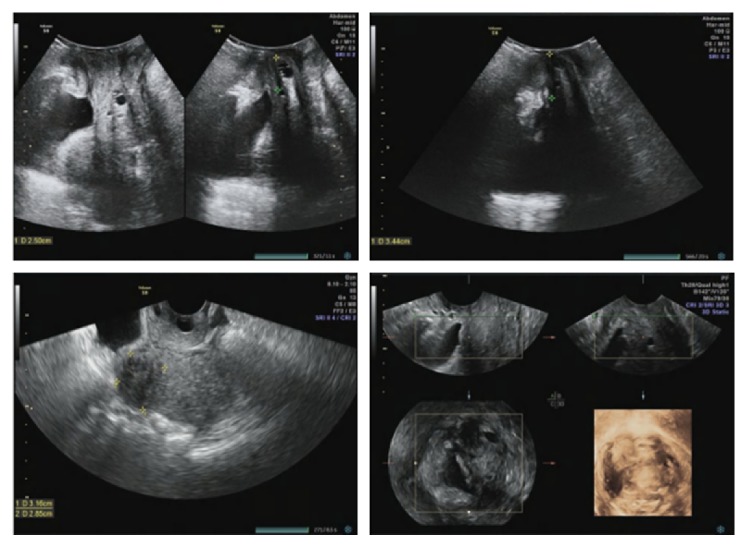
Pelvic floor four-dimensional ultrasound of Case 2. Pelvic floor four-dimensional ultrasound indicated that residual urine was 0 ml, thickness of detrusor was normal, internal orifice of urethra was closed, posterior angle of bladder was intact, and there was no dark area of liquid and scattered point of calcification around urethra in quiescent condition. CDFI revealed that sparse color flow signals were seen around the urethra, the bladder neck was 19 mm above the pubic symphysis, the uterus was 17 mm above the pubic symphysis, and ampulla portion of rectum was located at the pubic symphysis. Bladder neck displacement was 15 mm, bladder neck was located 9 mm below the pubic symphysis, posterior angle of bladder was intact, the uterus was 35 mm below the pubic symphysis, ampulla portion of rectum was located at the pubic symphysis, rectocele was not seen, and anal sphincter was complete in Valsalva.

The protrusion was not sensible while resting but rather palpable after moving. She visited our outpatient clinic at her 15th week of gestation in 2015 and complained worsened uterine prolapse. Pelvic examination revealed stage 3 POP, with point C as the leading edge using the POPQ examination (Aa+3, Ap+3, Ba+6, Bp+6, C+6, D+1, gh 5, pb 1, tvl 10 ). A no. 5 ring pessary in size of 7×7 cm (see Figure 1) was applied to keep the uterus inside the pelvic cavity after manual reposition. The gravid uterus persisted in the abdominal cavity after removing at the 30th week of gestation because it became larger. An alive healthy baby boy of 2680 g was delivered after four-hour labor at 39+3 week's gestation on the 5^th^ of October 2015. She was discharged three days postpartum with complete resolution of the uterine prolapse. A follow-up postpartum examination after 42 days revealed evidence of uterine prolapse and a no. 3 ring pessary in size of 5×5 cm has been applied to keep the uterus inside the pelvic cavity after manual reposition until now. At the time of reporting, pelvic examination of this woman revealed stage 3 POP, with point C as the leading edge using the POPQ examination (Aa-2, Ap-2, Ba-1, Bp-1, C+2, D-3, gh 5, pb 1, tvl 10). Pelvic floor four-dimensional ultrasound indicated that bladder neck mobility was slightly increasing, posterior wall of the bladder was slightly bulged, and anterior vaginal wall was slightly prolapsed in anterior compartment. Stage 2 uterus prolapse was seen in middle compartment, the levator animuscle was not broken, and hiatus of levator animuscle was normal in posterior compartment (see Figure 2). Follow-up is on-going.

## 3. Discussion

Uterine prolapse is a common case in nonpregnant older women; however, uterine prolapse complicating pregnancy is a rare event, which either exists before or has an acute onset during pregnancy.

The etiology of uterine prolapse during pregnancy is probably multifactorial. Parity, malnutrition, race, vaginal delivery, short interval between consecutive pregnancies, increased strain on the support of the uterus, physiologic change of pregnancy causing cervical elongation, hypertrophy and relaxation of the support ligament, and previous medical record of prolapse are among the most common risk factors [2]. Uterovaginal prolapse is more common in white and Hispanic women compared to women of African or Asian descent [3, 4].

POP presenting before pregnancy is less common and often resolves during pregnancy, but recurs after delivery [5–7]. Acute onset of POP in pregnancy is more common; it is usually firstly noted in the third trimester [5] and disappears after labor and delivery [8]. This might be due to a different aetiology compared with prepregnancy POP. This type of prolapse is most frequently caused by a history of trauma to the pelvic floor or congenital disorder that weakens the pelvic floor support. Prolapse developing in pregnancy is more likely to be due to an escalation of the physiological changes in pregnancy which lead to weakening of pelvic organ support [9]. Pregnancy itself may have triggered the prolapse. Increased cortisol and progesterone levels during pregnancy may contribute to the uterine relaxation. Damage to the genitourinary supports from repeated pregnancies and labor are the most important predisposing factors in POP. During childbirth, the pelvic floor is extended due to direct pressure of the fetal presenting part and maternal pressure effects. Decline in the elevator antimuscle tone is caused either by denervation or by direct muscle trauma, and hence resulting in an open urogenital hiatus, which, combining with the functional and anatomic alterations in the muscles and nerves of the pelvic floor, contribute to the development of POP. This would explain why the prolapse almost always recurs or persists in patients with prepregnancy prolapse, but spontaneously resolves in those developing during pregnancy. It would also explain the possible protective effect of a caesarean section in patients with acute onset of POP in pregnancy and not in those with prepregnancy POP [10].

The two patients in this report are multiparous women. Uterine prolapse during pregnancy most frequently occurs in multiparous women. None of the two patients in this report had uterine prolapse during the first pregnancy, but they had it in their second, even third pregnancy. Mant et al. [11] reported that women with twice vaginal deliveries have four times higher risk of prolapse compared to nulliparous women. Erata et al. [12] reported that the relative risk of developing uterine prolapse was 2.48 (95 % confidence interval [CI], 0.69–9.38) in women who had given birth to one child and increased to 4.58 (95 % CI, 1.64–13.77), 8.4 (95 %CI, 2.84–26.44), and 11.75 (95 % CI, 3.84–38.48) in women who had delivered 2, 3, or >3 children, respectively, compared with nulliparous women.

Uterine prolapse in pregnancy can cause antepartum, intrapartum, and puerperal complication. Antepartum complications include preterm labor, abortion, urinary tract infection, acute urinary retention, and even maternal death. The main intrapartum complications include inability to attain adequate cervical dilatation, as well as cervical laceration, obstructive labor, hysterorrhexis at the lower segment of the uterus, fetal death, and maternal morbidity. Puerperal infection and postpartum hemorrhage due to uterine inertia are common consequences of POP after delivery [13]. Similar to other case reports, our patients had antepartum complication of PROM, but we did not observe any intrapartum or puerperal complication. Moreover, Lau and Rijhsinghani [14] used Magnesium Solution to prevent cervical dystocia and lacerations for a prolapsed cervix which is edematous. We use Magnesium Sulfate Solution to nurse the prolapsed uterus postpartum in Case 1; the mechanism proposed may be due to osmotic diuretic properties of magnesium.

Successful pregnancy outcome requires individualized treatment with respect to patient's wishes, gestation, and severity of prolapse. Obstetrician should consider the above-mentioned possible complications. The management varies from a conservative approach to laparoscopic treatment. Conservative management with genital hygiene and bed rest in a moderately Trendelenburg position to enable prolapse replacement should be considered as the foremost treatment option. These precautions protect the cervix from trauma desiccation and reduce the incidence of preterm labor. Case 1 had successful pregnancy outcome because of bed rest. This again demonstrated that bed rest in a moderately Trendelenburg position is a practical management strategy.

Continual use of a pessary is recommended, which should not be removed until the onset of labor [6, 7]. A no. 5 ring pessary was applied to keep the uterus inside the pelvis after manual reposition and protect the prolapsed cervix in Case 2. The patient was managed with close follow-up on an outpatient basis. The gravid uterus persisted in the abdominal cavity because it became larger, and the pessary was removed at the 30^th^ week of gestation. In 1949, Klawans and Kanter [15] advised continual use of the Smith-Hodge pessary throughout the pregnancy for women with late occurrence of prolapse. Vaginal pessaries can be obtained and applied easily. Vaginal discharge, odor, mucosal erosion and abrasions of vagina, and urinary retention are common complications of vaginal pessaries [16]. For this patient, we did not encounter any of these complications. Different types of vaginal pessary have been used, but this management was reported as unsuccessful in literature since pessaries frequently fell out after a few days. Contrary to the literature, our case was managed successfully with a pessary. The ring pessary and its size perfectly fitted the patient. The patient was taught how to use the pessary and she performed the procedure perfectly. Thus, selection of pessary shape and its size and the patient's congruity to the treatment are the basis of success of this management.

When conservative management fails and prolonged bed rest is impossible, laparoscopic uterine suspension may be another treatment choice during early pregnancy. However, this procedure should be performed with experienced hands since several failed laparoscopic uterine suspension cases have been reported [17].

The method of delivery should be individualized according to the patients' preferences, status of cervix uteri, and labor progression. A vaginal delivery can be expected. Nonetheless, according to our experience, an elective caesarean section near term could be a valid and safe delivery option when the prolapsed uterus cannot be restored. Patient in Case 2 already had a favorable ripened cervix and the prolapsed uterus has already been in the pelvic cavity when she was referred to our hospital at 39+3 week's gestation. We did not have to insist on a caesarean section, so the patients ended with vaginal delivery. However, considering cervical dystocia, which results in inability to attain adequate cervical dilatation, in addition to obstructive labor, as well as cervical laceration and a predisposition to rupture of the lower uterine segment, emergency caesarean section was performed to avoid the above-mentioned intrapartum complication in Case 1.

Follow-up is necessary, pelvic floor four-dimensional ultrasound can clearly show the spatial relationship of anterior, middle, and posterior compartments in pelvic cavity, and pelvic examination and pelvic floor four-dimensional ultrasound may be a valid method for follow-up.

## 4. Conclusion

Obstetricians as well as all involved caregivers should be aware of this rare phenomenon, as early diagnosis is crucial for a safe gestation. Conservative treatment of these patients throughout pregnancy can lead to an uneventful, normal, spontaneous delivery. Management of uterine prolapse in pregnancy during labor should be individualized depending on the severity of the prolapse, gestational age, parity, and patient's preference.

## References

[1] De Vita D., Giordano S. (2011). Two successful natural pregnancies in a patient with severe uterine prolapse: A case report. *Journal of Medical Case Reports*.

[2] Tarnay C. M., Dorr C. H., DeCherney A. H., Nathan L. (2003). *Current Obstetric And Gynecology Diagnosis and Treatment*.

[3] Obed S. A., Kwawukume E. Y., Emuveyan E. E. (2005). Pelvic Relaxation. *Comprehensive Gynaecology in the Tropics*.

[4] Ugboma H. A., Okpani A. O., Anya S. E. (2004). Genital prolapse in Port Harcourt, Nigeria. *Nigerian Journal of Medicine*.

[5] Horowitz E. R., Yogev Y., Hod M., Kaplan B. (2002). Prolapse and elongation of the cervix during pregnancy. *International Journal of Gynecology and Obstetrics*.

[6] Hill P. S. (1984). Uterine prolapse complicating pregnancy. A case report. *The Journal of Reproductive Medicine*.

[7] Brown H. L. (1997). Cervical prolapse complicating pregnancy. *Journal of the National Medical Association*.

[8] Guariglia L., Carducci B., Botta A., Ferrazzani S., Caruso A. (2005). Uterine prolapse in pregnancy. *Gynecologic and Obstetric Investigation*.

[9] O'Boyle A. L., O'Boyle J. D., Calhoun B., Davis G. D. (2005). Pelvic organ support in pregnancy and postpartum. *International Urogynecology Journal*.

[10] Rusavy Z., Bombieri L., Freeman R. M. (2015). Procidentia in pregnancy: a systematic review and recommendations for practice. *International Urogynecology Journal and Pelvic Floor Dysfunction*.

[11] Cingillioglu B., Kulhan M., Yildirim Y. (2010). Extensive uterine prolapse during active labor: A case report. *International Urogynecology Journal*.

[12] Erata Y. E., Kilic B., Güçlü S., Saygili U., Uslu T. (2002). Risk factors for pelvic surgery. *Archives of Gynecology and Obstetrics*.

[13] Tsikouras P., Dafopoulos A., Vrachnis N. (2014). Uterine prolapse in pregnancy: Risk factors, complications and management. *The Journal of Maternal-Fetal and Neonatal Medicine*.

[14] Lau S., Rijhsinghani A. (2008). Extensive cervical prolapse duringlabor: a case report. *The Journal of Reproductive Medicine*.

[15] Klawans A. H., Kanter A. E. (1949). Prolapse of the uterus and pregnancy. *American Journal of Obstetrics & Gynecology*.

[16] Sulak P. J. (1997). Nonsurgical correction of defects, the use of vaginal support devices. *Te Linde’s Operative Gynecology*.

[17] Matsumoto T., Nishi M., Yokota M., Ito M. (1999). Laparoscopic treatment of uterine prolapse during pregnancy. *Obstetrics & Gynecology*.

